# Trade-off between thermal tolerance and insecticide resistance in *Plutella xylostella*

**DOI:** 10.1002/ece3.1380

**Published:** 2015-01-06

**Authors:** Lin Jie Zhang, Zhao Li Wu, Kuan Fu Wang, Qun Liu, Hua Mei Zhuang, Gang Wu

**Affiliations:** Key Laboratory of Biopesticide and Chemical Biology (Ministry of Education), Fujian Agriculture and Forestry UniversityFuzhou, 350002, China

**Keywords:** *Caspase*, fitness cost of chlorpyrifos resistance, heat shock proteins, *Plutella xylostella*, resistance-related enzymes, thermal tolerance

## Abstract

Fitness costs associated with resistance to insecticides have been well documented, usually at normal temperature conditions, in many insect species. In this study, using chlorpyrifos-resistant homozygote (R_R_) and chlorpyrifos-susceptible homozygote (S_S_) of resistance *ace1* allele of *Plutella xylostella* (DBM), we confirmed firstly that high temperature experience in pupal stage influenced phenotype of wing venation in insecticide-resistant and insecticide-susceptible *Plutella xylostella*, and S_S_ DBM showed significantly higher thermal tolerance and lower damages of wing veins under heat stress than R_R_ DBM. As compared to S_S_ DBM, R_R_ DBM displayed significantly lower AChE sensitivity to chlorpyrifos, higher basal GSTs activity and P450 production at 25°C, but higher inhibitions on the enzyme activities and P450 production as well as reduced resistance to chlorpyrifos under heat stress. Furthermore, R_R_ DBM displayed significantly higher basal expressions of *hsp69*s, *hsp72*s, *hsp20*,*hsp90*,*Apaf-1,* and *caspase-7* at 25°C, but lower induced expressions of *hsp*s and higher induced expressions of *Apaf-1*,*caspase-9,* and *caspase-7* under heat stress. These results suggest that fitness costs of chlorpyrifos resistance in DBM may partly attribute to excess consumption of energy caused by over production of detoxification enzymes and *hsp*s when the proteins are less demanded at conducive environments but reduced expressions when they are highly demanded by the insects to combat environmental stresses, or to excess expressions of apoptotic genes under heat stress, which results in higher apoptosis. The evolutionary and ecological implications of these findings at global warming are discussed.

## Introduction

Although resistance is a benefit to pests when they are treated with the agrochemicals, it is known that this resistance may come at a cost of other life-history traits. This fitness cost associated with resistance to agrochemicals has been well documented in many species (Bourguet et al. 2004; Berticat et al. 2008) and is believed to be the main constraint for the quick emergence of agrochemical resistance in agro-ecosystems. In insects, considerable fitness penalty in life-history and physiological traits associated with insecticide resistance has been found in *Plutella xylostella* (Sayyed and Wright 2001; Raymond et al. 2005; Cao and Han 2006; Liu et al. 2008), *Leptinotarsa decemlineata* (Zhu et al. 1996), *Anopheles gambiae* (Djogbénou et al. 2010), *Cydia pomonella* (Boivin et al. 2001, 2003; Konopka et al. 2012), *Culex pipiens quinquefasciatus* (Hardstone et al. 2009), *Myzus persicae* (Ghadamyari et al. 2008; Castañeda et al. 2011; Silva et al. 2012), *Aedes aegypti* (Martins et al. 2012), and *C. pipiens* (Lenormandm et al. 1999). Resource depletion is often assumed to be partially responsible for the observed fitness cost. Producing large amounts of detoxifying enzymes reduces energy availability for other biological and physiological functions. Perturbed physiological functions caused by excess production of detoxifying enzymes are also thought to be partially responsible for fitness costs (Higginson et al. 2005; Konopka et al. 2012).

Temperature is an important environmental factor that can exert critical influences on all biological, ecological, and evolutionary processes of species (Sørensen et al. 2003; Sørensen 2010). In insects, temperature can affect not only their species abundance and geographic distributions but also their interactions with other biotic and abiotic factors such as insecticides (Mpho et al. 2001; Chang et al. 2007). The majority studies on the interaction between insects and insecticides were conducted at conducive environments (Desneux et al. 2007) and changing environments such as temperature may play an important role on the evolutionary trajectory of the interaction as indicated in plant–pathogen system (Laine 2008). Changing temperature modifies the biological and genetic activities as well as behavior of insect species such as gene expressions, organ development, fecundity, respiration, and endocrine systems (Neven 2000), altering the responses of insects to insecticides. For example, functional properties of enzymes differing among temperature schemes in willow beetles (Rank et al. 2007) and wing morphology in *Drosophila* result from joint effects of mutations and development temperature (Debat et al. 2009).

The best-documented response of species to changing temperature at molecular level is the elicitation of heat shock proteins (Hsps). Most Hsps function as molecular chaperones by helping organisms to cope with heat stresses caused by either internally or externally and to eliminate themselves if the damages caused by the heat stresses become irreversible. A small amount of induced Hsps can have a major effect on development, resistance to stresses, longevity, and fecundity in many organisms including insects (Sørensen et al. 2003; Sørensen 2010). Among heat shock protein gene family (*hsp*s), *hsp70*s has been best characterized. Hsp70s are highly conserved and present in almost all species including bacteria, yeast, insects, vertebrate, plants, and mammals, suggesting their biological importance in protecting cells under stresses (Sørensen et al. 2003; Zhao and Jones 2012). However, overexpression of *hsp70*s may also pose a fitness cost to species. Positive correlations between *hsp70*s expression and thermal tolerance as well as the fitness costs of *hsp70*s overexpression have been documented in various insect species (Moseley 1997; Dahlgaard et al. 1998; Bahrndorff et al. 2009). In addition to Hsp70s*,* other Hsps with high or low molecular weight such as Hsp90s and Hsp20s, also play an important role in the physiological responses to heat stress (Frydenberg et al. 2003; Chen et al. 2005).

Apoptosis, triggered by heat stress (Jin et al. 2007; Hsu et al. 2011), is a conserved phenomenon widely involving in the reconstruction of multicellular organisms and elimination of old or damaged cells (Cho and Choi 2002; Sreedhar and Csermely 2004). Three main pathways of apoptosis include mitochondria-associated apoptotic pathway (intrinsic), death receptor pathway (extrinsic), and endoplasmic reticulum signal transduction pathway. Among them, mitochondria-associated apoptotic pathway is considered to be critically important. The mitochondria-associated apoptotic pathway includes the apoptotic signal transduction of apoptosome (consisted of cytochrome c-Apaf-1-procaspase-9) and the apoptotic executioner of caspase cascade (such as initiator caspase-9 at upstream and effector caspase-7, and-3 at downstream) (Cullen and Martin 2009). Effector caspases such as caspase*-*7 at the downstream of caspase cascade play the final execution of apoptosis in the apoptotic signaling pathways mediated by caspase (Cooper and Granville 2009; Zhuang et al. 2011). Many Hsps such as Hsp70s*,* Hsp20s, and Hsp90s have anti-apoptotic effects by inhibiting key steps in apoptotic cascade (Sreedhar and Csermely 2004; Didelot et al. 2006), but others such as Hsp60s may facilitate the apoptotic process (Beere et al. 2000; Tan et al. 2009).

*Plutella xylostella* (diamondback moth, DBM) is one of the most devastating pests worldwide and can multiply ∼18 generations each year in Fuzhou, Fujian Province, China (Wu and Jiang 2002). At the optimal temperature of 25°C, the life longevity of the insect is ∼20 days with ∼3 days each for the development of egg, one of four instar larva and pupa and 1–2 days for adult pre-oviposition (Shirai 2000; Liu et al. 2008). Current management of DBM relies primarily upon heavy applications of insecticides. Frequent and widespread uses of insecticides may lead to quick emergence of resistant insects, and insecticide resistance has been found in the field populations of DBM (Miyata and Wu 2010).

Chlorpyrifos, targeting to acetylcholinesterase (AChE) (a key enzyme regulating nervous system of insects), is an organophosphate insecticide used worldwide to control insect pests. It has been reported that reduced AChE sensitivity and increased GSTs activity and P450 production were responsible for the chlorpyrifos and other organophosphate resistance in DBM (Miyata and Wu 2010). Three amino acid mutations (A201S, G227A, and A441G) were found in insecticide-resistant DBM. A201S and G227A were located at the AChE active site and were thought to be responsible for organophosphates resistance in DBM (Baek et al. 2005; Lee et al. 2007). Field surveys between 1999 and 2007 revealed that resistance to insecticides declined sharply over summer in the field DBM populations from Fuzhou, but recovered to a high level in spring and autumn (Wu and Jiang 2002; Liu et al. 2008). LC_50_ values to insecticides methamidophos, dichlorvos, methomyl, carbofuran, fenvalerate, cypermethrin, avermectin, and chlorfluazuron, which are belonged to several different classes of insecticides, in summer DBM populations were 20–30% of those in the spring or autumn populations (Wu and Jiang 2002; Liu et al. 2008). Control experiments also showed resistance to methamidophos, and avermectins declined sharply after the DBM insects were reared at 33.5°C for one generation (Liu et al. 2008; Zhuang et al. 2010). These results suggest that insecticide-susceptible DBM have significant advantages in life-history traits under heat stress than insecticide-resistant DBM. However, this observation has not been confirmed experimentally. It is also not clear what are the mechanisms responsible for a low fitness of chlorpyrifos resistance in DBM under heat stress.

However, the fitness costs of insecticide resistance were studied based on evaluations of life-history and/or physiological traits, usually. The literature dealt with the damages in morphological phenotype as the evidences of the fitness costs of insecticide resistance in insects could not be found to date. The second, although the fitness cost associated with resistance to agrochemicals has been well documented in many insects; however, the majority studies on this field were conducted at the temperature, which was conducive to the survival and reproduction of insects. The study on the effects of high temperature on the fitness costs of insecticide resistance was limited. The last, it was not clear that how the responses in expressions of *hsp*s and apoptosis-relevant genes and toxicological characteristics to heat stress in both insecticide-resistant and insecticide-susceptible insect strains.

The goal of this study was to provide the evidence between fitness costs of insecticide resistance in biological, physiological, or toxicological characteristics, in particular in morphological damages of wing veins as affected by heat stress and to investigate the mechanisms responsible for the evolutionary trade-off between insecticide resistance and thermal adaptation in DBM. Using chlorpyrifos-resistant DBM (R_R_, resistant homozygote population at all of three amino acid mutations of *ace1* allele) and chlorpyrifos-susceptible DBM (S_S_, susceptible homozygote population at the three amino acid mutations of *ace1* allele), we ask (1) whether the members of Hsps family and mitochondria-associated apoptotic pathway gene family were involved in the evolution of thermal adaptation in DBM by determining the mRNA expression profiles of the genes; (2) whether and how the fitness cost of insecticide resistance under heat stress existed in DBM by comparing the biological, physiological, and toxicological performances.

## Materials and Methods

### Experimental populations

A DBM population was collected from the commercial crucifer fields located at Shangjie, Fujian, China, 20 kilometers away from Fujian Agriculture and Forestry University (FAFU), in November 2005. No specific permissions were required for our collection of DBM, because the scientists were welcome to collect the insect sample from the farmer's crucifer fields to control the pest insects. The field studies did not involve endangered or protected species. The DBM was subsequently trained on *Brassica oleracea* in an insecticide-free field insectarium at FAFU for 1 year (about 18 generations). Eight hundred pupae each were randomly chosen from the trained population in November 2006 and reared separately in two field insectariums (A and B). The insectariums (4 m × 2 m × 4 m) were made of stainless-steel net with a glass roof to prevent the contamination of insects from external DBM populations. Insects in insectarium A were not treated with any insecticides and were highly susceptible to chlorpyrifos after November 2008, hereafter defined as chlorpyrifos-susceptible population (S_i_). In insectarium B, the insects had been treated with 48% chlorpyrifos EC (commercial name: Dursban) since November 2006. The population of DBM in insectarium B included different developmental stages (i.e., 1st instar larva to 4th instar larva, pupa, and adults) and was selected on the basis of chlorpyrifos resistance. The insect population derived from this insectarium after 24 months (November 2008) was highly resistant to chlorpyrifos and hereafter defined as chlorpyrifos-resistant population (R_c_). Susceptible genotype (S_S_) was created by crossing a male and female insect randomly chosen from S_i_ population and resistant genotype (R_R_) was generated by treating R_c_ population several generations with a dose of chlorpyrifos that resulted in ∼97% DBM mortality at 25°C. The S_S_ and R_R_ were then maintained in separate field insectariums. R_R_ (100% *RR* at A201S and G227A) and S_S_ (100% *SS* at A201S and G227A) were confirmed by molecular assay of *ace1* as described previously (Baek et al. 2005; Lee et al. 2007). Among the three amino acid mutations detected in *ace1* sequence (GenBank: JQ085429, JQ085428), the frequency of A201S and G227A mutants was positively correlated with chlorpyrifos resistance in DBM (*r* = 0.74, df = 62, *P *<* *0.0001), and this result was derived from molecular and phenotypic analyses of 64 artificial populations composed of more than 2850 insect individuals (GW, DB, LJZ, YPJ, XHL, CWL unpubl. data), consisting with the hypothesis that A201S and G227A were responsible for the chlorpyrifos resistance in DBM. The difference in chlorpyrifos resistance between R_R_ and S_S_ was >100-fold. The 4th instar larvae, pupae or adults aged within 4–8 h were used for all experiments unless specifically defined.

### Effects of high temperature on activity of detoxifying enzymes

The adults emerged at 25°C were used to assay AChE, CarE, and GST activities, and P450 production. AChE, CarE, and GST activities were assayed using the protocol described previously (Wu and Miyata 2005), and P450 production was quantified with an extinction coefficient of 91 mmol\L^−1^ cm^−1^ based on the difference in absorbance at 450 nm and 490 nm (Omura and Sato 1964). The insects collected from the field insectarium were reared at 25°C under insecticide-free condition, and F_1_ progenies were used for assays. The insects were reared at 36 and 40°C for 1, 4, 8, and 24 h, respectively, before they were used for these assays. In control experiment, the insects were reared at 25°C for same time. The enzyme assay was replicated at least three biological replicates with 10 insect individuals in each replication. About 70% RH were used in this study unless specifically defined.

### Effects of temperature on resistance to chlorpyrifos

Ninety-five percent technical grade chlorpyrifos (Shandong Huayang Technology, Shandong, China, Co. Ltd.) was used for bioassays. Bioassay of insecticide resistance was conducted with or without high temperature pretreatment using vial dry film (Liu et al. 2008). In the experiment with high temperature pretreatment, the adults emerged at 25°C were first reared in an insecticide-free vial at 36 and 40°C for 24 and 8 h, respectively, and then removed to a glass vial covered with dry-chlorpyrifos-film at 25°C for bioassay of resistance to chlorpyrifos. Insect mortality was recorded at 24, 48, and 72 h after they were kept in contact with the insecticide. In the control experiment (without high temperature pretreatment), the adult insects emerged at 25°C were first reared in an insecticide-free vial at 25°C for 8 or 24 h, and then used for bioassay. Acetone solution of chlorpyrifos was used to prepare a glass vial dry film (1.2 cm × 10 cm). The concentrations of chlorpyrifos used for the experiment were 1667 mg/L (=LC_35_), 1000 mg/L (=LC_18_), and 500 mg/L (=LC_5_) for R_R_ DBM and 50 mg/L (=LC_74_), 25 mg/L (=LC_45_), and 12.5 mg/L (=LC_18_) for S_S_ DBM. The three doses of chlorpyrifos were calculated from toxicity regression equation causing about 10, 30, and 50% mortality of R_R_ and S_S_ adults, respectively.

### Effects of high temperature on pupa survival, adult emergence, and hind-wing venation

**Figure 1:**
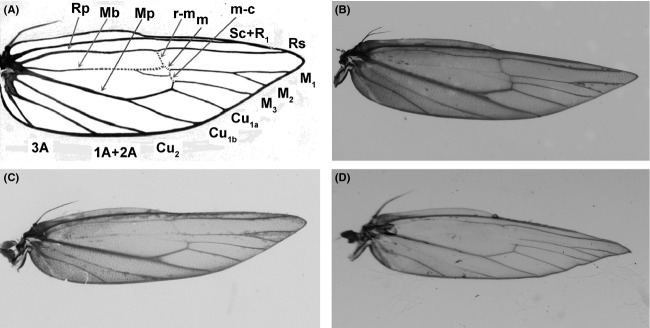
Schematic hind-wing venation and possibly missing veins in DBM. In panel A, Sc: Subcosta; R_1_: 1st Radial vein; Rs: Radial sector; M_1_-M_3_: 1st to 3rd Media; Cu_1_ and Cu_2_: 1st and 2nd Cubitus; 1A+2A: 1st Anal+2nd Anal; 3A: 3rd Anal; r-m: Radiomedial crossvein; m: Medial crossvein; m-c: Medialcubitus crossvein; c: Cubitus crossvein; Rp: Radinus Posterior; Mb: Medial bar; Mp: Medial Posterior. The missing vein meant the vein disappeared and could not been seen. The whole of r-m, m or m-c, or the front part of Mb (indicated by dot line) might be missed individually or concurrently, after the pupae were treated with 38°C for 48 h, 40°C for 8 or 16 h, 42°C for 4 or 8 h, or 44°C for 1 h, respectively. B was unmissing veins, C and D were missing veins.

In our previous research, we found heat stress resulted in damages of DBM's wing veins. Because the wings develop inside the pupae as wing buds, we speculated that the experience of high temperature during pupal stage was important for the development of adult's wings. Pupae were then used for heat stress treatments. R_R_ and S_S_ DBM were reared at 25°C until pupae were formed. The pupae were divided into six groups and incubated at 38°C for 48 h, 40°C for 8 and 16 h, 42°C for 4 and 8 h, and 44°C for 1 h, respectively, before they were placed back to 25°C for growth and development. These temperature schemes were selected to allow enough insects survival from the heat stress and in the meanwhile to reflect the true temperature fluctuation in Fuzhou during the hottest season (between July 10 and August 10) in a year. In the hottest day during this period of time, day temperature in Fuzhou can reach at 38–40°C for ∼6–8 h, 40–42°C for ∼2–3 h, and 42–44°C 1–2 h in the fields. Pupae, which did not respond to pencil tip prodding after heat treatments, were believed to be dead, and survival rates of pupae (the percentage of pupae alive) were recorded after high temperature treatments. The rates of adult emergence were recorded 3 days after incubation at 25°C. Hind wings of emerged adults were collected, and the scales of wing were rinsed out using 70% alcohol so that the veins could be seen clearly. The proportion of DBM with damaged wings was recorded. Earlier studies showed that wing sizes and shapes in *Drosophila* could be affected by temperatures (Debat et al. 2009). However, it is not clear whether heat stress may have different effects on wing venation of insecticide-resistant and insecticide-susceptible insects. Wing damage was thought to happen when one or more veins of hind wing were missed. The missing vein meant the vein disappeared and could not been seen as indicated by dot line in Fig.1, that is, vein r-m, m and m-c, or Mb which altered hind-wing venation. The whole of r-m, m or m-c vein, or the front part of Mb vein could be missed individually or concurrently after the pupae were treated with high temperature. Two hind wings from each adult insect were used to determine the rate of wing damage because the damage might occur in one or both hind wings at any adult insect. As soon as one of the veins among r-m, m or m-c vein, or the front part of Mb vein was disappeared in a wing, the vein damage was thought to be occurred. Survival rates of pupae and rates of adult emergence were 100%, and rates of wing vein damage were zero when the pupae were incubated at 25°C for the same time. About 300–500 pupae with same age were included in the estimates of pupa survival and adult emergence rates, and ∼75–300 adults (or about 150–600 hind wings) were included for the estimate of wing damage rate in each assay. Because large sample size (insect number) increased statistical robustness of experimental results, we included as many insect individuals as possible in the experiments. To reduce environmental heterogeneity, same insect individuals were kept in each vial, resulting in 7–11 replicates in the measurements of pupae survival, adult emergence, and wing venation.

### Inducing thermal tolerance of adults

The adults emerged at 25°C were incubated at 37°C for 3 h. The heat-treated adults were placed back to 25°C for 1 h for recovery and incubated at 45°C for another 1 h before being moved back to normal growth temperature of 25°C. Survival rate of the insects was recorded 24 h after the second heat stress treatment. The heat stress assay was replicated five times with 30 insect individuals in each replication.

### Effects of high temperature on the expression of heat shock proteins (Hsps) and apoptotic pathway genes

A total of 12 heat treatments were included in this analysis. Corresponding to six pupa treatments described in the experiment to determine the effects of high temperature on pupa survival, adult emergence, and hind-wing venation, six pupae with same heat treatments were used to mRNA determinations. In addition, the adult's thermal tolerance was studied because the wing's developments would last after adult's emergence. Another six adult treatments were also included for determinations of *hsp*s and mitochondria-associated apoptotic pathway genes expressions. In the adult treatments, DBM emerged at 25°C were incubated at 25, 33.5, 37, 42, 47, and 50°C for 3 h, respectively. 33.5°C was chosen because the life-table parameters of both insecticide-resistant and insecticide-susceptible DBM were significantly different at this temperature in our previous experiments (Liu et al. 2008). Fourty-seven and 50°C were chosen to determine the response of DBM to the extreme high temperature. After heat stress treatment, the survival rates were about 50–90% for pupae, and 100% (at or lower than 42°C for 3 h), about 70% (at 47°C for 3 h), or 40% (at 50°C for 3 h) for adults. Living pupae or adults after heat stress were allowed to recovery for 1 h at 25°C before they were used for detecting mRNA expression.

The mRNA expressions of *hsp*s and apoptotic pathway genes were detected by real-time quantitative PCR (qPCR) using SYBR Premix Ex Taq ™ kit (Takara Co., Dalian, China). The total RNA was isolated by RNA simple total RNA extract kit (Tiangen Co., Bejing, China), and cDNA was synthesized by PrimeScript ™ reagent kit (Takara Co.). The *hsp*s used for qPCR included *hsp69-1* (GenBank: ADK94697.1), *hsp69-2a* (GenBank: ADK94698.1), *hsp69-3* (GenBank: ADK94699.1), *hsp69-4* (GenBank: ADK39311.1), *hsp72-2* (GenBank: ADV58254.1), *hsp72-3* (GenBank: ADV58255.1), *hsc70* (GenBank: JN676213), *hsp90* (GenBank: KF471526), and *hsp20* (GenBank: KF471527) obtained by us in DBM of Fujian, China and *hsp72-J* (GenBank: BAF95560.1) identified by a Japanese team (Sonoda and Tsumuki 2008), respectively. Among them, the *hsp69*s, *hsp72-J,* and *hsp72*s were named as *hsp70*s hereafter. Different *hsp69*s or *hsp72*s were named depending on their molecular weight (varied from 69.00 to 69.27 kDa or from 72.39 to 72.58 kDa). The mitochondria-associated apoptotic pathway genes included *caspase-7* (GenBank: HM204505) (Zhuang et al. 2011), c*ytochrome c* (GenBank: KC507801), *Apaf-1* (GenBank: KC588901), and *caspase-9* (GenBank: KF365914), which were also identified by us in DBM of Fujian, China. The primers used for qPCR were designed from the conserved regions of the genes (Table S1). mRNA transcriptions were quantified using the protocol described previously (Zhuang et al. 2011) with following PCR conditions: 95°C for 10 s, followed by 40 cycles of 95°C for 6 s, 60°C for 25 s. Fluorescence was measured after each cycle. The homogeneity of the PCR products was confirmed by melting curve analysis. Relative mRNA expression of *hsp*s and mitochondria-associated apoptotic pathway genes was measured in reference to the housekeep gene *β*-actin, which was amplified by PCR using primers *β*-actin-F and *β*-actin-R (Table S1) (Zhuang et al. 2011). The transcriptional level of inner-control was similar among different samples. The mean normalized expression of *hsp*s and mitochondria-associated apoptotic pathway genes was calculated by comparing the threshold cycle of these genes to that of *β*-actin gene according to the equations of standard curves of the target genes and the reference gene (Larionov et al. 2005). The standard curves of target genes and reference gene were made using 10 times serial dilutions with six different cDNA concentrations. The mRNA expression was replicated at least three biological replicates with 12 insect individuals in each replication.

### Data analyses

LC (lethal concentration) values of chlorpyrifos and toxicity regression equation were calculated from the bioassay of chlorpyrifos resistance using a DPS data processing system (Tang and Feng 1997). The difference in chlorpyrifos resistance between R_R_ and S_S_ DBM was calculated by taking a ratio of their respective LC_50_. Enzyme inhibition was calculated by taking ratio of activity index of the enzyme in each treatment and in control. Duncan's multiple range tests and *t*-tests (Milton and Arnold 1995) were used to compare phenotypic (rates of pupae survival, adult emergence, wing damage, resistance-related enzymes, and susceptibility to chlorpyrifos) and genotypic (gene expressions) between R_R_ and S_S_ insects and among temperature treatments using the same DPS data processing system (Tang and Feng 1997).

## Results

### Effects of high temperature on enzyme activity and AChE sensitivity to chlorpyrifos

**Figure 2:**
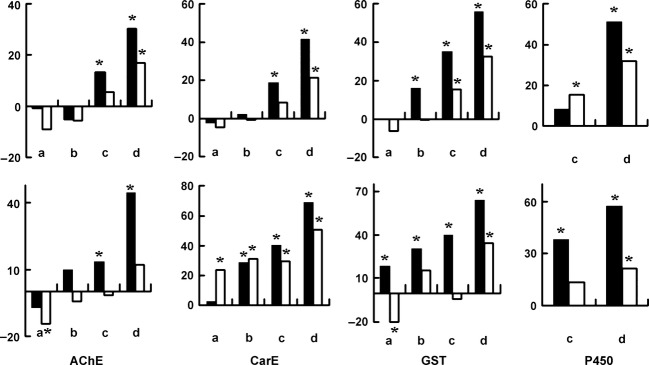
The inhibitions on enzyme activity and P450 content in adults of S_S_ (white) or R_R_ (black) under heat stress. The control was reared at 25°C. Ordinate indicated the inhibition rate of enzyme activity or P450 content. Asterisk indicated significant differences between treatment and control in S_S_ or R_R_ (*t*-test, *P *≤ 0.05). The insects were reared at 36°C (upper panels) or 40°C (bottom panels) for 1(a), 4(b), 8(c), and 24(d) h, respectively, before the adults were used for biochemical assays. Enzyme inhibition was calculated by taking ratio of activity index of the enzyme in each treatment and in control.

**Figure 3:**
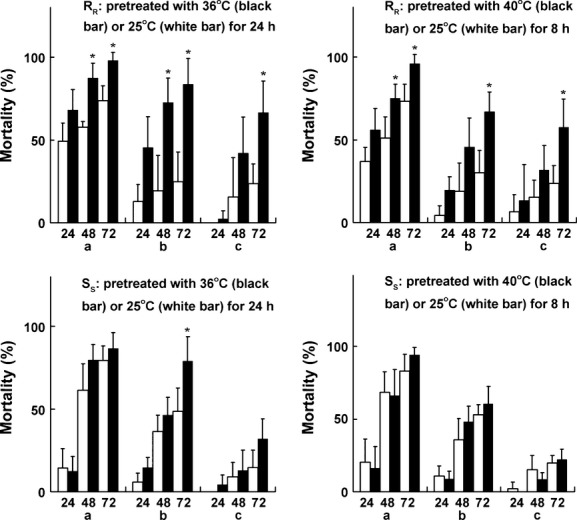
Effects of high temperature pretreatments on the susceptibility of R_R_ (up two panels) and S_S_ (down two panels) adult DBM to chlorpyrifos. Black bars: the adults were pretreated with 36 and 40°C for 24 and 8 h, respectively under insecticide-free condition at the first and then used for bioassay of chlorpyrifos resistance at 25°C. The mortality was recorded at 24, 48, and 72 h after the insects were treated with the insecticide, respectively. White bars: the controls (without high temperature pretreatment). Lower letters (a, b, and c) in abscissa indicate the doses of chlorpyrifos used in the experiment, that is, 1667 (a), 1000 (b), and 500 mg/L (c) for R_R_ DBM, and 50 (a), 25 (b), and 12.5 mg/L (c) for S_S_ DBM, respectively. Asterisk indicates a significant difference in mortality (*P *≤ 0.05) between the high temperature pretreatments (black bar) and the control (white bar) (*t*-test).

As compared to S_S_ DBM, R_R_ DBM showed significantly lower AChE sensitivity to chlorpyrifos (the lower the *k*_i_ value of AChE was, the lower the sensitivity to chlorpyrifos was) and higher basal GSTs activities and P450 production at 25°C (Table1). The activities of the four resistance-related enzymes were inhibited in all heat treatments in both S_S_ and R_R,_ but the inhibitions were significantly higher in R_R_ than in S_S_ (Fig.2). Although R_R_ showed significantly higher GSTs activities and P450 production than S_S_ at 25°C, the two groups of insects displayed similar GSTs activities and P450 production at 36°C and 40°C for 8 or 24 h. Under heat stress, the susceptibility of R_R_ adults to chlorpyrifos at doses of LC_35_, LC_18,_ and LC_5_ increased significantly in terms of the mortality at 48 and/or 72 h. This pattern was not observed in S_S_ adults (Fig.3).

**Table 1:** *k*_i_, AChE, CarE, and GST activities, and P450 content in R_R_ and S_S_ DBM

DBM	*k*_i_ ± SE* *× 10^2^	AChE (nmol/mg protein 20 min)	CarE (nmol/mg protein)	GST (×10^3^ nmol/mg protein min)	P450 (nmol/mg protein)
S_S_ larva	–	39.1 ± 1.8a	60.1 ± 3.7a	62.7 ± 3.3a	0.057 ± 0.001a
R_R_ larva	–	37.2 ± 0.9a	44.9 ± 1.6b	84.6 ± 7.9b	0.127 ± 0.012b
S_S_ adult	(6.73 ± 0.33)	52.6 ± 2.4a	15.2 ± 0.2a	40.2 ± 0.2a	0.0313 ± 0.0031a
R_R_ adult	(0.48 ± 0.05)	54.6 ± 2.5a	18.7 ± 0.6b	60.7 ± 2.2b	0.048 ± 0.011b

Notes: The insects collected from the field insectarium were reared at 25°C under insecticide-free condition, and F_1_ progenies were used for assays. Data followed by different letters in the same column indicate significant difference at *P *≤* *0.05.

### The effects of heat stress on pupae survival, adult emergence, and hind-wing venation

**Figure 4:**
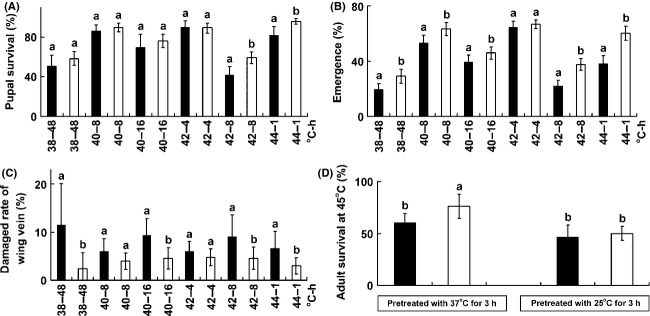
Thermal tolerance of R_R_ (black) and S_S_ (white) DBM. The survival rate of pupae (A) was recorded at the first. The pupae were then removed to 25°C and the emergence rate of adult (B) was recorded several days later. The proportion of adults with damaged wing veins (C) was then recorded. The adult mortality at 45°C for 1 h (D) was recorded after the insects were pretreated at 37 or 25°C for 3 h. Abscissa in (A–C): temperature (˚C)-treated time (h), indicating after the newly pupae were pretreated with high temperature for a given time (i.e., 38°C for 48 h, 40°C for 8 or 16 h, 42°C for 4 or 8 h, or 44°C for 1 h, respectively). Lower-case letter indicates significant difference between R_R_ and S_S_ DBM (*t*-test, *P* ≤ 0.05).

There was no difference in survival rates of R_R_ and S_S_ pupae reared at 38°C for 48 h, 40°C for 8 or 16 h, and 42°C for 4 h, but significantly higher survival rates were observed in S_S_ than R_R_ pupae at 42°C for 8 h and 44°C for 1 h (Fig.4A). Adult emergence rates were significantly higher in S_S_ than R_R_ under all temperature treatments except 42°C for 4 h (Fig.4B). Among the emerged adults, damages to the hind-wing venation in the two hind wings were significantly severe in R_R_ genotypes than S_S_ genotypes in all treatments except 40°C for 8 h and 42°C for 4 h (Fig.4C). Although survival rates at 45°C increased when insects were pretreated with a higher (37°C) than lower (25°C) temperature, significantly higher survival rates were observed only in S_S_ adults (Fig.4D).

### Expression of *hsp*s and apoptotic pathway genes under heat stresses in pupae

**Figure 5:**
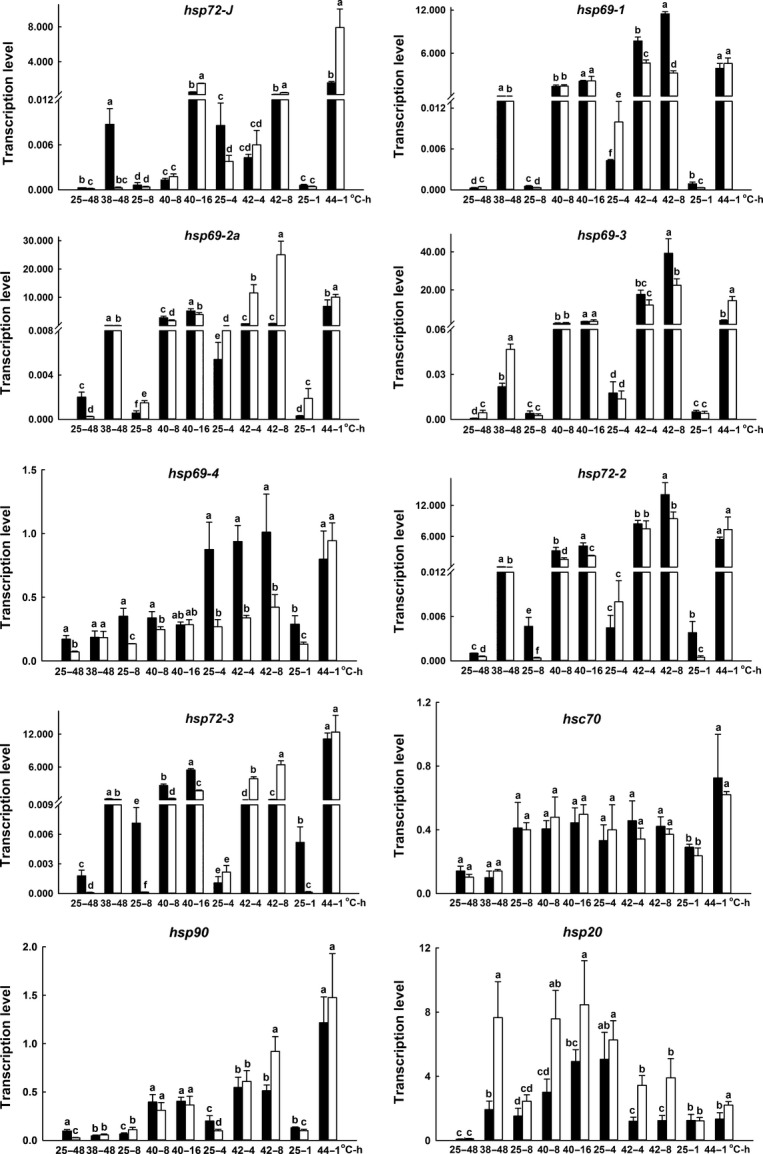
Effects of heat stress on the expression of *hsp70s*,*hsc70*,*hsp90,* and *hsp20* in R_R_ (black) and S_S_ (white) pupa DBM. Ordinate: relative expression of the genes. Abscissa: temperature (˚C)-treated time (h). Pupae newly formed at 25°C from both R_R_ and S_S_ populations were incubated by four temperature treatments, that is, 25 or 38°C for 48 h (25–48 or 38–48), 25 or 40°C for 8 (25–8 or 40–8) or 16 h (40–16), 25 or 42°C for 4 (25–4 or 42–4) or 8h (42–8), and 25 or 44°C for 1 h (25–1 or 44–1), respectively. Living pupae after heat stress were allowed to recovery for 1 h at 25°C before they were used for detecting mRNA expression. The values in the each group of the four temperature treatments were used for statistic analysis, respectively, in R_R_ and S_S_ DBM. Lower-case letter indicates significant difference in mRNA expression in each temperature treatment group (Duncan test, *P *≤ 0.05).

**Figure 6:**
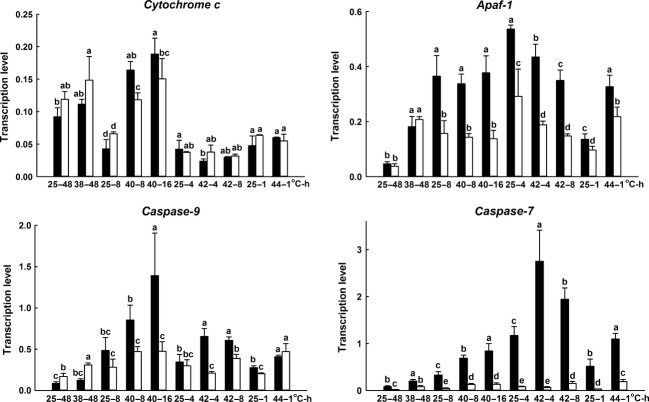
Effects of heat stress on the expression of mitochondria-associated apoptotic pathway genes in R_R_ (black) and S_S_ (white) pupa DBM. Ordinate: relative expression of the genes. Abscissa: temperature (˚C)-treated time (h). Pupae newly formed at 25°C from both R_R_ and S_S_ populations were incubated by four temperature treatments just as those described in Fig. 5, and living pupae after heat stress were used for detecting mRNA expression. The data in each temperature treatment group were tested by Duncan test (*P* ≤ 0.05).

**Figure 7:**
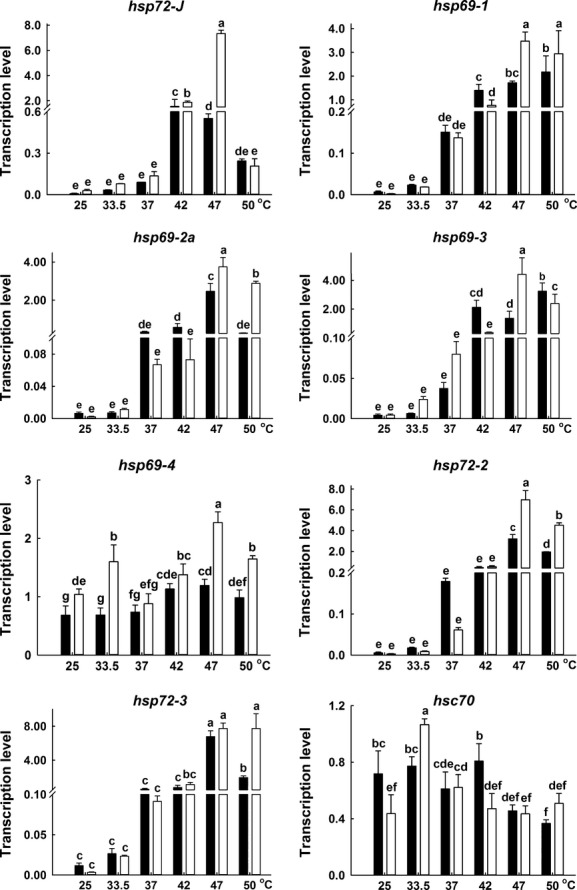
The expression profiles of *hsp70s* in R_R_ (real line) and S_S_ (broken line) adult DBM under extreme high temperature for 3 h. Ordinate: relative expression of the genes. Abscissa: temperature (˚C). The adults were used for assays after the insects were reared at 25, 33.5, 37, 42, 47, or 50°C for 3 h, respectively. Different lower-case letter indicates significant difference in mRNA expression among different temperature treatments in each gene (Duncan test, *P *≤ 0.05).

Among the four temperature treatment groups, that is, 25°C or 38°C for 48 h, 25°C or 40°C for 8 or 16 h, 25°C or 42°C for 4 or 8 h, and 25°C or 44°C for 1 h, no significant upregulated expression of *hsc70* under heat stress were observed in S_S_ and R_R_ pupae. The basal mRNA expression of *hsp70*s (i.e., *hsp72-J*,*hsp69-1*,*hsp69-2a*,*hsp69-3*,*hsp69-4*,*hsp72-2,* and *hsp72-3*) were lower than that of *hsc70* at 25°C but their upregulated expressions under heat stress were higher than *hsc70* in both pupae and adults of S_S_ and R_R_ genotypes (Figs5 and 6). S_S_ pupae displayed significantly lower basal expressions of *hsp70*s and *hsp90* but higher upregulated expressions of the genes than R_R_ pupae under heat stress with some exceptions (Fig.5). For instance, S_S_ genotypes displayed significantly higher basal expression in *hsp69-1* and *hsp69-2a* at 25°C for 4 h and *hsp90* at 25°C for 8 h (Fig.5) than R_R_ genotypes. On the other hand, no significant upregulation expression of *cytochrome c* under heat stress in S_S_ and R_R_ genotypes except at 40°C for 8 and 16 h, and no great differences in expression of *cytochrome c* between S_S_ and R_R_ genotypes were found whatever at 25°C or high temperature conditions, in general. As compared to 25°C treatment, 38°C for 48 h, or 44°C for 1 h resulted in significant upregulation expression of *Apaf-1* in S_S_ and R_R_ genotypes. However, 40°C for 8 or 16 h and 42°C for 4 or 8 h did not result in upregulation expression of *Apaf-1* in S_S_ and R_R_ genotypes. In addition, expressions of *Apaf-1* in R_R_ genotypes were significantly higher than those in S_S_ at 25°C or heat stress except for 25°C or 38°C for 48 h. Significant upregulation expression of *caspase-9* under heat stress in S_S_ and R_R_ genotypes was found, but the extent of the upregulation was significantly higher in R_R_ genotypes than S_S_ genotypes at 40°C for 8 and 16 h, and 42°C for 4 and 8 h. Regarding to *caspase-7*, S_S_ genotypes showed significantly lower basal expression at 25°C, compared to R_R_ genotypes. The expression of *caspase-7* was induced greatly by heat stress in R_R_ genotypes and significantly higher than S_S_ genotypes, but there were no significant upregulation expression of *caspase-7* in S_S_ genotypes under heat stress, in general (Fig.7).

### Expression of *hsp*s and apoptotic pathway genes in adults under heat stresses

**Figure 8:**
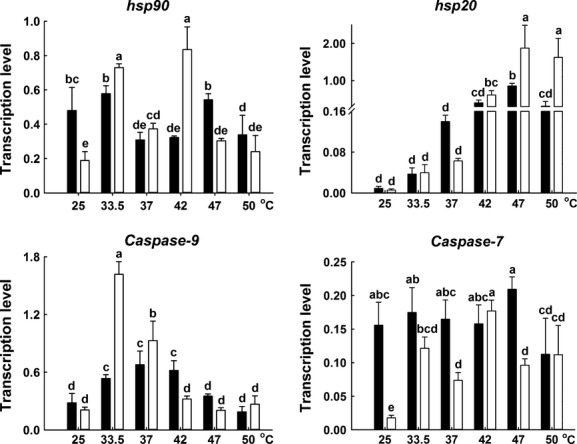
The expression profiles of *hsp90*,*hsp20* and apoptotic pathway genes in R_R_ (black) and S_S_ (white) adult DBM under high temperature for 3 h. The adults were used for assays after the insects were reared at 25, 33.5, 37, 42, 47, or 50°C for 3 h, respectively. Different lower-case letter indicates significant difference in mRNA expression among different temperature treatments in each gene (Duncan test, *P *≤* *0.05).

S_S_ adults displayed similar or significantly lower basal expressions of *hsp*s than R_R_ adults (Figs6 and 8). The expressions of *hsc70* oscillated among different temperature treatments, and there were no differences in the expressions between S_S_ and R_R_ adults in general. However, the expressions of *hsp69*s, *hsp72*s, *hsp20,* and *hsp90* increased with the initial increase of temperature, reached at a plateau at 47°C and then decreased at 50°C, in general. Although the expression pattern in some genes oscillated among different temperature treatments, compared to R_R_ genotypes, S_S_ genotypes displayed significantly higher expressions of *hsp69*s, *hsp72*s, *hsp20,* and *hsp90* in general, and S_S_ genotypes displayed lower expressions of *caspase-7* at extreme high temperatures (47°C) (Figs6 and 8).

## Discussion

### Hsp70 family and evolution of thermal adaptation

Total nine *hsp70*s (including 5 *hsp69*s and 4 *hsp72*s), one *hsc70*,*hsp90,* and *hsp20* were identified from DBM in China, and previous studies indicated that Hsp70 proteins were encoded by a multigene family in some other insect species, such as *Drosophila melanogaster* (Mou et al. 2011) and *Anopheles albimanus* (Benedict et al. 1993). Although in some cases, there existed a lack of correlation between *hsp*s expression and thermotolerance in several cases, positive correlation between *hsp*s expression and induced thermotolerance, or between *caspase-7* expression and mortality under heat stress were found in S_S_ and R_R_ or R_c_, in general. Multiple gene family and upregulated expression were considered to be two important mechanisms in species adaptations (Bettencourt et al. 2002). Multiple *hsp*s would be contributive to produce a deal of Hsps in a short time under stress. In addition, the genes might be regulated by different mechanism to meet different cellular needs during growth and development process under varied environmental conditions.

### Fitness cost of insecticide resistance under heat stress in DBM

We found that S_S_ DBM displayed higher biological and physiological fitness than R_R_ DBM under heat stress condition. S_S_ DBM showed higher survival and adult emergence rates than R_R_ DBM (Fig.4). S_S_ DBM also showed lower wing-damaged rate than R_R_ DBM. Wing-damaged under higher temperature is thought to be caused by changing venation of insects during pupal developments and is a common phenomenon in insect species (Debat et al. 2009). In honey bees, physical structures and behavioral performances of adult were influenced by the temperature at pupal development (Tautz et al. 2003). Our results are consistent with previous findings.

To avoid the potential effects of other than insecticide-resistance traits on biological and physiological performance of DBM, S_S_ and R_R_ populations used in our study were descended directly from the same starting population. In the three *ace1* mutants, A201S and G227A were confirmed to be responsible for the resistance to chlorpyrifos (GW, unpubl. data) and other organophosphate insecticides (Baek et al. 2005; Lee et al. 2007). S_S_ was 100% susceptible homozygote (*SS*) at A201S and G227A, and R_R_ was 100% resistant homozygote (*RR*) at A201S and G227A. Therefore, our results indicated that the individual with *SS* genotype (in S_S_) showed lower damages of DBM's wing veins than *RR* genotype (in R_R_).

Our results provide clear evidence on the evolutionary trade-off between insecticide resistance and thermal adaptation in DBM and are consistent with previous studies showing that the resistance to insecticide resulted in considerable disadvantages in life-history traits at both normal (Sayyed and Wright 2001; Raymond et al. 2005; Cao and Han 2006) and high temperatures (Liu et al. 2008) in DBM and other pest insect species (Hardstone et al. 2009; Djogbénou et al. 2010; Konopka et al. 2012; Martins et al. 2012; Silva et al. 2012). The trade-off between insecticide resistance and thermal adaptation may explain why resistance to organophosphates, carbamates, pyrethroids, benzoylurea, and *Bt* in the field populations of DBM declined sharply during hot summer but was maintained at a higher level during spring and autumn (Wu and Jiang 2002; Liu et al. 2008).

### Molecular mechanisms of trade-off between insecticide resistance and thermal adaptation in DBM

Trade-off between insecticide resistance and thermal adaptation in DBM may partly attribute to the differences in the expression of *hsp*s and other genes between *RR* genotype (in R_R_) and *SS* genotype (in S_S_). Compared to *SS*,*RR* genotypes showed lower AChE sensitivity (i.e., lower *k*_i_ value) and higher activities of detoxification enzyme (GST and P450) at 25°C when there was no chlorpyrifos presence (Table1). There are two types of heat shock genes in organisms. *hsp* is stress-inducible, and *hsc* is constitutively expressed gene. The “housekeeping” function of Hsps in cells under conducive environment was performed by constitutive expressions. Under stress conditions, the requirements for “housekeeping” and other functions were increased and were supplemented by the induction of additional expression. Mutations conferring resistance to insecticides may cause constitutive fitness costs when a large amount of resources from the insects is diverted to its resistance mechanisms (Table1) (Konopka et al. 2012). In this study, the key members of Hsp family and mitochondria-associated apoptotic pathway genes were studied. It was shown the different profiles of *hsp*s and apoptosis-related gene expressions acted as a critical coordinator in deciding the differences in apoptosis and thermal tolerance under heat stress in insecticide-resistant and insecticide-susceptible DBM (Figs7). R_R_ DBM displayed higher basal *hsp*s and *caspase*-*7* expression at normal temperature and lower upregulated *hsp*s expression and higher upregulated *caspase-7* expression under heat stress than S_S_ DBM. In addition, R_R_ DBM displayed higher upregulated expression of *Apaf-1* and *caspase-9* in some of high temperature treatments than S_S_ DBM. Elevated basal *hsp*s and *caspase-7* expression in R_R_ DBM at 25°C could be a penalty for growth and development of insects because additional amounts of resources are allocated to translate the proteins. On the other hand, decreased expressions of *hsp*s and enhanced expressions of *Apaf-1*,*caspase-9,* and *caspase-7* at high temperature reduce the ability of R_R_ DBM to stand up for heat stress or increased their apoptotic process. The positive relationship between *hsp*s expression and biological performance such as fecundity was also found in *Chrysomela aeneicollis* (Dahlhoff et al. 2008).

There have been a great number of researches using the expressions of heat shock genes to infer natural adaptation of species (Sørensen 2010) because the genes can act as a capacitor of phenotypic variation and evolution (Feder and Hofmann 1999; Christine et al. 2002). The individuals that cannot produce enough heat shock proteins could be disadvantage in nature (Sørensen 2010). However, overexpressions of heat shock genes may have a negative impact on growth, development, and fertility of species. The fitness cost of overexpressing heat shock genes are thought to be associated with the shutdown of normal cell functions during the stress response, the extensive use of energy, and the toxic effects of high concentration of heat shock proteins interfering normal cell function (Hoffmann 1995; Sørensen et al. 2003; Kristensen et al. 2008). For example, it has been shown that overexpressions of heat shock proteins can retard growth, cell division and fecundity of species (Krebs and Feder 1997; Silbermann and Tatar 2000; Dahlhoff et al. 2008). These results suggest that natural selection favors for genotypes with a balanced production of heat shock proteins (Hoffmann 1995; Sørensen et al. 2003; Kristensen et al. 2008; Sørensen 2010).

The results indicated that insecticide-resistant DBM showed significant disadvantages in the evolutionary response to heat stress. We provided firstly an evidence, that is, more severe damages in morphological phenotype of wing veins as fitness costs of insecticide resistance as affected by heat stress, and confirmed firstly that the fitness costs of chlorpyrifos resistance in DBM may partly attribute to excess consumption of energy caused by over production of detoxification enzymes, *hsp*s and *caspase-7* when the proteins are less demanded at conducive environments, but reduced expressions of *hsp*s when they are highly demanded by the insects to combat environmental stresses, or increased expressions of apoptosis-related genes, which resulted in more severe apoptosis. This finding has important implications in controlling DBM during global warming. Insecticides are the primarily approach for DBM and other pest management in agricultural ecosystem. Frequent applications of insecticides may select for DBM with high resistance and the resistant DBM may spread quickly to other populations by migration, leading to widespread of resistant DBM and a quick loss of insecticide used to control the insects over large geographic areas. Increase in air temperature during global warming may slow down the emergence of DBM with high insecticide resistance, increasing the lifespan of insecticides and reducing the costs of controlling DBM, and other important insects in agriculture. When designing insect management program, seasonal fluctuation in temperature should be considered to maximize the effect of insecticides and minimize costs and residues of controlling insects.

## Data Archiving Statement

All of the raw data underlying the main results of the study were archived in Fujian Agriculture and Forestry University, Fujian Province, China.

Data deposited in the Dryad repository: The sequences of *hsp69*s, *hsp72*s, *hsc70*,*hsp90*,*hsp20*,*cytochrome c*,*Apaf-1*,*caspase-9,* and *caspase-7* were stored in GenBank.

## Supporting Information

Table S1. Primer sequences used for qPCR of *hsp69*s, *hsp72*s, *hsc70*,*hsp90*,*hsp20*,*cytochrome c*,*Apaf-1*,*caspase-9* and *caspase-7*.
