# Supplementary material for: Trade-off between thermal tolerance and insecticide resistance in Plutella xylostella
Source: Ecol Evol. 2015 Jan 6;5(2):515–30. doi: 10.1002/ece3.1380 (PMC4314281; doi:10.1002/ece3.1380)
Supplement: Supplementary file 1 — Table S1. Primer sequences used for qPCR of hsp69s, hsp72s, hsc70,hsp90,hsp20,cytochrome c,Apaf-1,caspase-9 and caspase-7. [file ece30005-0515-sd1.doc]

Supporting information

**Table S1. Sequences of primers for real-time quantitative PCR (qPCR)**

| Names of Primers | Sequences of Primers | Gene names |
| --- | --- | --- |
| β-actin-F^*^ | 5'-ACCGGTATCGTGCTGGACTC-3' | *β*-actin |
| β-actin-R^*^ | 5'-GCCATCTCCTGCTCGAAGTC-3' |
| Pxh1-F | 5'-CTGCTGGTGGATGTGGCT-3' | *hsp69-1* |
| Pxh1-R | 5'-TGGTTGTCCGCGTAGGTC-3' |
| Pxh2-F | 5'-CGGCATCGACTACTACACCA-3' | *hsp69-2a* |
| Pxh2-R | 5'-GCCTCCGACTAAGACCACAT-3' |
| Pxh3-F | 5'-GCGTACCTCGGGACTACTG-3' | *hsp69-3* |
| Pxh3-R | 5'-TGGGCTCGTTGATGATGC-3' |
| Pxh4-F | 5'-TCGCCTTCACCGACACC-3' | *hsp69-4* |
| Pxh4-R | 5'-TTGCCTCCATCACTGACCAC-3' |
| Pxh5-F | 5'-CGACGGCATCGACTACTACA-3' | *hsp72-2* |
| Pxh5-R | 5'-GAGAGCCTTTTCAACGGGTT-3' |
| Pxh6-F | 5'-CGAAGCGAATTAAAACCA-3' | *hsp72-3* |
| Pxh6-R | 5'-TCCGTGAAAGCCACATAT-3' |
| Pxhj-F | 5'-GCGAAGCAAGAACATCGTGA-3' | *hsp72-J* |
| Pxhj-R | 5'-AGTTTGTCCCCGGATTCCTC-3' |
| Pxhsc70-F | 5'-CTCCGTATTATCAACGAACC-3' | *hsc70* |
| Pxhsc70-R | 5'-CACCTCCCAAGTGAGTGTCT-3' |
| q20-F | 5'-GCCCATCTCCCACACAG-3' | *hsp20* |
| q20-R | 5'-CAAGCGAACGACCCTCA-3' |
| q90-F | 5'-AGTTCATCGGCTACCCC-3' | *hsp90* |
| q90-R | 5'-TCGTCCTCACCCTCCTC-3' |
| qCytc-F | 5'-CCCAGTGCCACACAGTTG-3' | *cytochrome c* |
| qCytc-R | 5'-GCCTTCTTCAGACCAGCG-3' |
| qApaf-1F | 5'-CTTCTGGCTGAACTTGGGC-3' | *Apaf-1* |
| qApaf-1R | 5'-GCTAGGGAATGGCTGTCGT-3' |
| qCas-9-F | 5'-TCGCGTTCAGCTTCATCG-3' | *caspase-9* |
| qCas-9-R | 5'-CAGCGTGTCTTCTGTCTCCTTC-3' |
| qCas-F | 5'-CCCACTACAA GCCTGAC-3' | *caspase-7* |
| qCas-R | 5'-ATACGGCACTGGAAGAG-3' |
